# Interaction Studies of Hexameric and Pentameric IgMs with Serum-Derived C1q and Recombinant C1q Mimetics

**DOI:** 10.3390/life14050638

**Published:** 2024-05-17

**Authors:** Maria Magdalena John, Monika Hunjadi, Vanessa Hawlin, Jean-Baptiste Reiser, Renate Kunert

**Affiliations:** 1 Institute of Animal Cell Technology and Systems Biology, Department of Biotechnology, BOKU University, Muthgasse 11, 1190 Vienna, Austria; maria.john@boku.ac.at (M.M.J.);; 2 Institut de Biologie Structurale, UMR 5075, University Grenoble Alpes, CNRS, CEA, 38000 Grenoble, France

**Keywords:** recombinant human IgM, C1q, C1q mimetics, protein interactions, complement activation, ELISA, bio-layer interferometry

## Abstract

The interaction between IgM and C1q represents the first step of the classical pathway of the complement system in higher vertebrates. To identify the significance of particular IgM/C1q interactions, recombinant IgMs were used in both hexameric and pentameric configurations and with two different specificities, along with C1q derived from human serum (sC1q) and two recombinant single-chain variants of the trimeric globular region of C1q. Interaction and complement activation assays were performed using the ELISA format, and bio-layer interferometry measurements to study kinetic behavior. The differences between hexameric and pentameric IgM conformations were only slightly visible in the interaction assay, but significant in the complement activation assay. Hexameric IgM requires a lower concentration of sC1q to activate the complement compared to pentameric IgM, leading to an increased release of C4 compared to pentameric IgM. The recombinant C1q mimetics competed with sC1q in interaction assays and were able to inhibit complement activation. The bio-layer interferometry measurements revealed K_D_ values in the nanomolar range for the IgM/C1q interaction, while the C1q mimetics exhibited rapid on and off binding rates with the IgMs. Our results make C1q mimetics valuable tools for developing recombinant C1q, specifically its variants, for further scientific studies and clinical applications.

## 1. Introduction

The complement system is an essential part of both innate and adaptive immune defense and plays a key role in the initial immune response against pathogens [1,2]. The classical pathway, which is one of the main activation pathways of the complement system, is strongly triggered by immunoglobulins M and G (IgMs, IgGs) once bound to their cognate antigens [3].

As the first component of the classical pathway, C1q recognizes, among other distinct structures, the Ig-constant regions and activates the complement cascade [4]. Dysregulation of C1q has been identified in various diseases, including several autoimmune disorders, such as systemic lupus erythematosus, increased susceptibility to infection when C1q is underrepresented, and inflammatory diseases when C1q is overrepresented [2,5].

The current structural activation model of the classical complement cascade suggests that IgMs undergo large conformal changes during binding to their antigens by the translocation of their Fab-Cµ2 regions from planar and highly mobile conformations to bent and fixed structures [6,7]. These structural changes in IgMs enable the exposure of a cryptic motif from Cµ3 domains, the foreseen binding sites of the C1q globular heads. Once the multimeric C1 complex is bound, activation of downstream substrates initiates the well-known proteolytic cascade [8]. As a result of complement activation, an immune response is initiated and the IgM targets are eliminated [9].

The interaction between the globular head parts of C1q and the Fc-regions of IgM was often studied by inhibition studies [10,11], but differences between hexameric and pentameric conformations of IgM have so far been little investigated [6,12,13].

In this present study, we evaluated the interactions between different recombinant IgMs, C1q, derived from human serum (sC1q), and two single-chain variants of the trimeric globular region of C1q based on the concept by Moreau et al. [14]. The recombinant IgMs were obtainable in two specificities and were expressed with and without the J-chain to generate IgMs in either pentameric (5IgM) or hexameric (6IgM) configuration [15]. We performed interaction assays, along with complement activation assays in the ELISA format, in a direct and competitive way. Bio-layer interferometry measurements were also used to evaluate protein interaction kinetics and affinities.

While only minor differences in interaction parameters between the IgMs and sC1q were observed with BLI, the hexameric IgMs activated the C4 cleavage 2.1 to 4.6 times more effectively than their pentameric counterparts. The C1q mimetics were able to inhibit the interaction of IgMs and sC1q as well as the activation of the complement.

Our research paves the way for the development of recombinant C1q, specifically variants thereof, for further scientific studies and in clinical application.

## 2. Materials and Methods

All chemical substances, unless otherwise specified, were purchased from Carl Roth GmbH + Co., KG (Karlsruhe, Germany).

### 2.1. Protein Design

The design of the C1q mimetics was based on the idea of Moreau et al. [14] to express the globular head of the C1q molecule as a single-chain version. Thus, the first globular construct, ACB, contains the three globular peptide chains of C1q joined by short linkers to form a single-chain protein (human C1qA residues 115–245, UniProtKB P02745; GSG-linker; human C1qC residues 87–217, UniProtKB P02747; GSA-linker; human C1qB residues 117–253, UniProtKB P02746 [16]).

For the second C1q mimetic, the method of Joung et al. [17] was used and further developed as in Lobner et al. [18]. This construct, ACB-AD2 (AD2), consists of the first variant (ACB) fused at the C-terminus of the B-chain via a glycine–serine linker to the domain 2 of the human serum albumin tag (human albumin residues 211–403, UniProtKB P02768). Both variants were equipped with a signal peptide (MDRAKLLLLLLLLLLPQAQA) [19] and a FLAG-tag (DYKDDDDK) [20] bound to the N-terminus of the A-chain for detection and purification. The theoretical molecular mass of the ACB protein backbone is 47 kDa and, in the case of AD2, it is 69 kDa, both carrying one N-glycosylation site in the A-chain of C1q (UniProtKB P02745 [21]).

### 2.2. Recombinant Cell Lines

All coding regions for the recombinant proteins were codon-optimized for chinese hamster ovary (CHO) cells, synthesized chemically and the CHO cell lines were developed in our laboratories.

The two DNA constructs for the artificial C1q mimetics were cloned into a pL vector (pCaggs promotor [22]) and CHO-K1, the host cell line (ATCC CCL-61), was transfected with Attractene (QIAGEN GmbH, Hilden, Germany) according to the manufacturer’s instructions. Clone selection and subcloning were achieved by limited dilution and 0.5 mg/mL of G418 (Geneticin disulfate solution) was used as the selection agent. Cells were routinely passaged every 3–4 days with HyClone ActiPro medium (Cytiva Europe GmbH, Freiburg, Germany) supplemented with 8 mM of Roti-Cell Glutamine solution, 15 mg/mL of Phenol red solution (Merck KGaA, Darmstadt, Germany and 1:500 diluted Anti-Clumping Agent (Thermo Fisher Scientific Inc., Waltham, MA, USA), which also served as a cultivation medium in all of the experiments.

For the recombinant IgMs, antibody-expressing recombinant CHO clones with two different specificities, HB617 and 2G12, were created. For each of these antibodies, two different CHO cell lines were established for the expression of either pentameric or hexameric IgM. For the pentameric conformation, the vectors for the heavy, light and joining chains were transfected in CHO-DG44 host cell lines (Thermo Fisher Scientific Inc., Waltham, MA, USA) [23]. For the hexameric conformation, the heavy and light chain constructs for IgM HB617 and IgM 2G12 were cloned into pcDNA3.1(+) vectors and subsequently transfected into CHO-K1 host cell lines [24].

### 2.3. Protein Production and Purification

To produce the recombinant C1q single-chain mimetics, the cells were cultivated in 200 mL shaking flasks, starting with 1 × 10^6^ cells/mL in 50 mL of cultivation medium for 72 h. After this initial batch phase, the cells were centrifuged (350× *g*, 7 min) and cultivation was continued as a semi-continuous perfusion process [25] with a daily medium exchange. After centrifugation, the supernatant was harvested, and the cells were resuspended in 50 mL of fresh medium. The process was run until the viability dropped below 80%. The supernatant was purified with Anti-DYKDDDDK G1 Affinity Resin (GenScript Biotech Corporation, Piscataway, NJ, USA) according to the manufacturer’s small-scale instructions. Briefly, 150 µL of slurry was washed with 1 mL of TBS and the supernatant discarded. All centrifuge steps were performed at 6000× *g* for 30 s. An amount of 900 µL of harvested supernatant along with 100 µL of 10× TBS was centrifuged at 6000× *g* for 10 min and, subsequently, the supernatant was transferred to the washed resin. After rotating the tubes for 1 h at room temperature, the resin was centrifuged for 30 s, the supernatant was discarded and the resin was washed three times with 1 mL of TBS. Elution was performed for a minimum of 5 min and a maximum of 15 min using 450 µL of Tris-glycine at pH 3.5. The pH of the purified protein was adjusted with 18 µL of 1 M Tris, pH 9.0. The resin was then washed three times with 1 mL of TBS, followed by a further round of purification using the same resin. The protein was purified twice, then combined and concentrated in a Vivaspin 6 5000 MWCO PES (Sartorius AG, Goettingen, Germany) and re-buffered with TBS. Its concentration was measured by Nanodrop (Thermo Fisher Scientific Inc., Waltham, MA, USA).

All IgMs were produced in the same manner as C1q mimetics using the semi- perfusion cultivation. The supernatants were purified according to the established protocol with the POROS CaptureSelect IgM affinity matrix (Thermo Fisher Scientific Inc., Waltham, MA, USA) described in Hennicke et al. [26].

### 2.4. SDS-PAGE

The SDS-PAGE was conducted using a NuPAGE 4–12% Bis-Tris, 1.0 mm Mini Protein Gel and run in NuPAGE MOPS SDS Running Buffer (1×). Reduced protein samples were prepared with NuPAGE Sample Reducing Agent (10×) (all NUPAGE reagents from Thermo Fisher Scientific Inc., Waltham, MA, USA). PNGase F (New England Biolabs GmbH, Frankfurt am Main, Germany) was used for following the non-denaturating protocol as recommended by the manufacturer. PageRuler Unstained Protein Ladder (Thermo Fisher Scientific Inc., Waltham, MA, USA) was used as a marker. All protein samples were mixed with NuPAGE LDS Sample Buffer (4×) (Thermo Fisher Scientific Inc., Waltham, MA, USA), and then incubated at 70 °C while shaking at 600 rpm for 10 min. They were then separated on the gel at 200 V for 45 min and silver-stained.

### 2.5. IgM/sC1q Interaction Assay

All assays were conducted using F96 Maxisorp Nunc-Immuno plates (Thermo Fisher Scientific Inc., Waltham, MA, USA). The four IgMs were coated with 50 µL of 2 µg/mL IgM in carbonate coating buffer and left overnight at 4 °C. PBS buffer containing 0.1% Tween was used for each washing step, and each incubation step was performed at room temperature with shaking at 400 rpm for 1 h. The plate was saturated using blocking buffer (a washing buffer solution with 2% polyvinylpyrrolidone (PVP, Merck KGaA, Darmstadt, Germany)). All sample dilution steps were performed with sample dilution buffer (TBS with 2% PVP, 5 mM of CaCl_2_ and 1.5 mM of MgCl_2_). For the interaction assay, 20 µg/mL of serum-derived human C1q (sC1q, Biosynth AG, Staad, Switzerland) was serially diluted as a sample. For the competitive interaction assay, 40 µg/mL of ACB or 20 µg/mL of AD2 were serially diluted and 30 µL of each C1q mimetic dilution was mixed with 30 µL of 20 µg/mL sC1q dilution. Then, 50 µL of each sample was applied to the IgM-coated plate. Detection was carried out using 50 µL of 1 µg/mL anti-C1qC/C1qG HRP polyclonal antibody (Bioss Inc., Woburn, MA, USA) diluted in blocking buffer. The colorimetric reaction was achieved with 100 µL of TMB substrate (Thermo Fisher Scientific Inc., Waltham, MA, USA) and stopped after approximately 5 min by adding 100 µL of 2.5 M H_2_SO_4_. A Tecan Spark multimode microplate reader (Tecan Group Ltd., Maennedorf, Switzerland) was used to measure OD at a wavelength of 450 nm with a reference wavelength of 620 nm. For normalization, the absorbance of the highest OD value was used as 100% in each assay. To estimate the relative IC_50_ for the competitive interaction assay, the OD value of 40 µg/mL of ACB or 20 µg/mL of AD2 for each IgM curve was used as 100% and the OD value without ACB or AD2 as 0%.

### 2.6. Complement Activation Assay

The complement activation of IgM was assessed using an ELISA based on the C4b deposition with minor modifications [24,27,28]. The four IgMs were coated with 50 µL of 10 µg/mL IgM in a Carbonate coating buffer and kept at 4 °C overnight. The washing steps, incubation steps and saturation of the plate were performed as above. All sample and serum dilution steps were performed with serum dilution buffer (TBS with 5 mM of CaCl_2_ and 1.5 mM of MgCl_2_). For the activation assay, 16 µg/mL of sC1q was serially diluted as a sample. For the competitive activation assay, 32 µg/mL of ACB was mixed with 1 µg/mL or 2 µg/mL of sC1q. Then, 30 µL of each sample dilution was combined with 30 µL of 12.5-fold diluted C1q-depleted human serum (Complement Technology, Inc., Tyler, TX, USA), and 50 µL of the resulting mixture was applied to the plate. In total, 50 µL of goat anti-human C4 antibody (1 µg/mL) (Complement Technology, Inc., Tyler, TX, USA) diluted in blocking buffer was used as first antibody which was detected with 50 µL of anti-goat IgG Peroxidase antibody (0.05 µg/mL) (Thermo Fisher Scientific Inc., Waltham, MA, USA), diluted in blocking buffer. An amount of 100 µL of TMB substrate (Thermo Fisher Scientific Inc., Waltham, MA, USA) was added as substrate and the reaction was stopped after roughly 5 min with the addition of 100 µL of 2.5 M H_2_SO_4_. Finally, the OD was measured using the Tecan Spark multimode microplate reader at a wavelength of 450 nm with a reference wavelength of 620 nm.

### 2.7. Bio-Layer Interferometry

Bio-layer interferometry (BLI) measurements were performed using the Octet RED96e instrument with Octet Protein L (ProL) Biosensors (Sartorius AG, Goettingen, Germany). TBS containing 2% PVP, 2 mM of CaCl_2_ and 0.02% Tween-20 was used as an assay buffer. The IgMs were diluted to 30 µg/mL, sC1q (Complement Technology, Inc., Tyler, TX, USA) ranged from 20 to 100 nM, ACB ranged from 196 to 976 nM and AD2 ranged from 133 to 667 nM. Regeneration was performed with glycine buffer at pH 1 for 30 s. The assay buffer was used to establish the baseline for 60 s before and after capturing the IgM for 500 s, further referred as IgM loading. Association lasted for 300 s, followed by 300 s of dissociation. An assay buffer was used as a reference and subtracted for further calculations. k_on_, k_off_ and K_D_ values were evaluated using Data Analysis HT software (version 11.1.1.39) through a global fit and a fast 2:1 heterogeneous ligand model.

## 3. Results

### 3.1. Production and Purification of the C1q Mimetics

**Figure 1:**
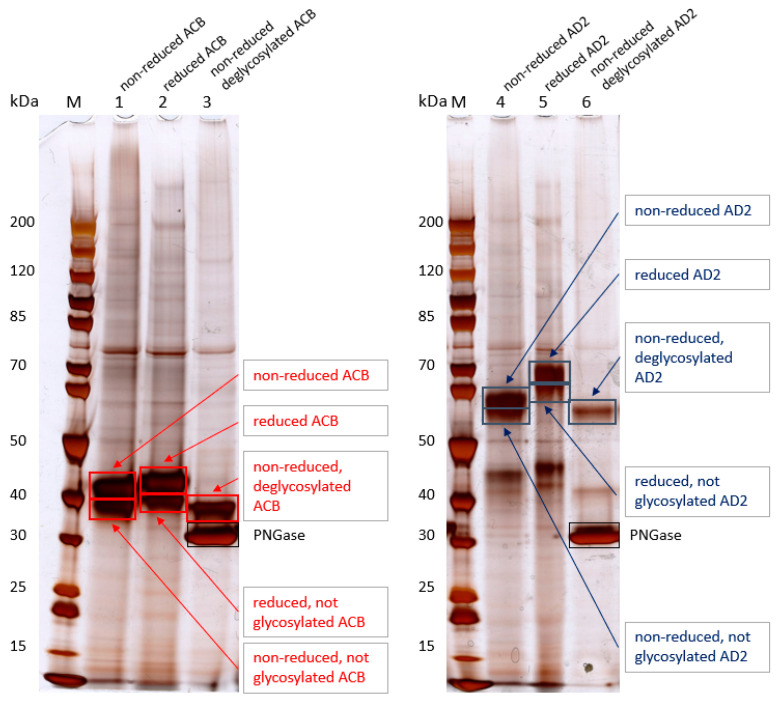
SDS-PAGE: lane 1–3, C1q mimetic ACB; lane 4–6, C1q mimetic AD2; lane M, PageRuler Unstained Protein Ladder; lane 1 and 4, protein under non-reduced conditions; lane 2 and 5, protein under reduced conditions; lane 3 and 6, protein under non-reduced and deglycosylated conditions.

The C1q mimetics ACB and AD2 were secreted from stably transfected CHO-K1 cells. The supernatant was collected every 24 h and subsequently purified using an affinity resin coated with an anti-FLAG monoclonal antibody. The purified recombinant proteins were concentrated, rebuffered and analyzed with SDS-PAGE. Figure 1 depicts the silver-stained gel displaying both C1q mimetics applied under either non-reduced, reduced or non-reduced and deglycosylated conditions.

The C1q mimetic ACB displayed two bands under non-reduced conditions (Figure 1, lane 1). The upper band approximated a molecular weight of ~45 kDa and the second band just below this. Because only one glycosylation site is found, the A-chain (human C1qA residue Asn^146^, UniProtKB P02745 [21]) and an additional ~2–3 kDa molecular weight is thus expected for ACB constructs; the upper band is attributed to the glycosylated form of ACB and the lower band to the non-glycosylated form of ACB. The glycosylation forms were also confirmed under deglycosylated conditions (Figure 1 lane 3), with the presence of a single band observed at the identical migration level as the lower band in lane 1. The bands with the reduced ACB in lane 2 appeared slightly higher than the non-reduced bands, which is attributed to the more open protein structure.

The molecular weight of the second C1q mimetic, AD2, exceeds that of ACB as the protein construct was fused with the human albumin domain 2, resulting in a glycoprotein of about 70 kDa. SDS-PAGE analyses exemplified the successful expression with one main band observable under reduced conditions (lane 5). Lane 4 exhibited a second, marginally lower band under non-reduced conditions, which could be attributed to the unglycosylated form and further confirmed under non-reduced and deglycosylated conditions (lane 6).

Some additional bands were still observable at a height of about 75 kDa in all samples and at about 45 kDa in the AD2 samples. Compared to the C1q mimetics, the amount of the byproduct was marginal, and the purity was considered sufficient for the following experiments. Thus, several milligrams of purified protein could be purified for each C1q mimetic with a concentration range of 0.1 to 0.2 mg/mL.

### 3.2. Interaction of IgM and sC1q Analyzed by ELISA

**Figure 2:**
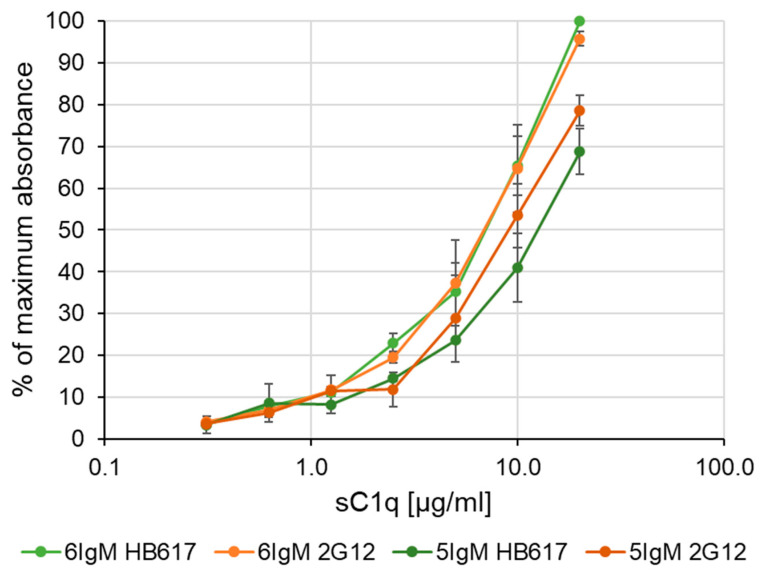
IgM/sC1q interaction measured by ELISA. Standard deviation was calculated from two individual assays. For normalization, the absorbance of the highest OD value was used as 100% in each assay.

An ELISA was used to evaluate the binding of sC1q to the different IgMs. Two IgMs with two distinct antigen specificities were produced: HB617, which targets the membrane glycosphingolipid of tumor cells GM3/GD3, and 2G12, which targets mannosic chains of the envelop protein of the human immunodeficiency virus (HIV) gp120 [29,30,31,32]. Each IgM was also applied in two configurations either pentamers (5IgM) or hexamers (6IgM). All four IgMs were then used as coated ligands. Serum-derived C1q (sC1q) represents the analyte and was detected with an anti-C1q HRP antibody. Normalized absorbance from two individual duplicated assays is shown in Figure 2.

Binding curves were generated with the four different IgM coatings and showed an sC1q concentration-dependent increase in signals. Both curves from the two hexameric IgM coatings showed the same behavior with a steeper increase in the signal and the highest final absorbance, while both curves from the pentameric IgM coatings reached 70–80% of the maximum signal at the highest sC1q concentration.

The steeper slope of the 6IgM binding curves and the maximum absorbance reached at the highest sC1q concentration indicates a slightly better binding of C1q to the 6IgMs compared to the 5IgMs.

### 3.3. Competition between sC1q and C1q Mimetics in a Competitive ELISA

To measure the binding of the C1q mimetics to coated IgMs in an ELISA format, anti-FLAG or anti-HSA antibodies were used in preliminary experiments. However, reliable results could not be achieved with this ELISA setup. One explanation might be the rapid dissociation of ACB and AD2 from coated IgMs (as shown below with BLI), which might prevent their detection. Therefore, the binding of ACB and AD2 to coated IgMs was measured indirectly using a competitive ELISA format.

**Figure 3:**
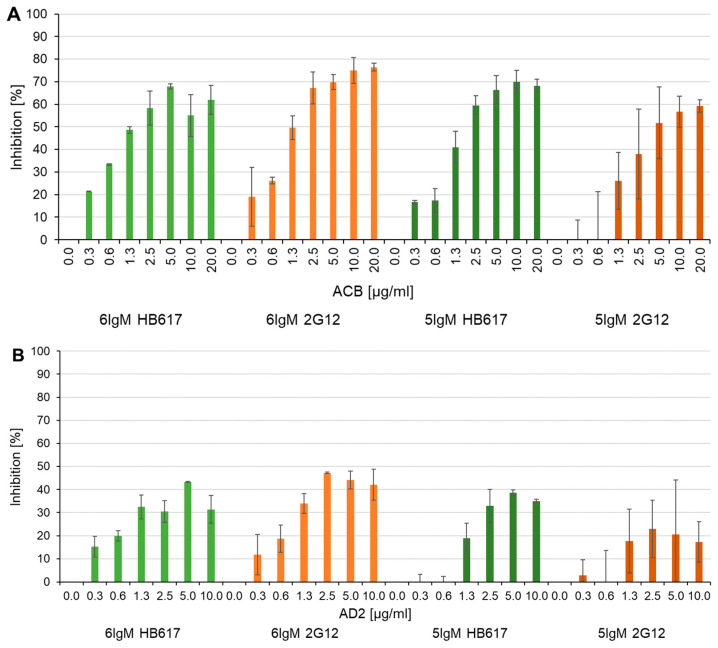
Inhibition of IgM/sC1q interaction measured by competition ELISA with IgM coating and anti-C1q detection. Four different IgMs were used for coating. Each IgM was tested with serially diluted ACB (**A**) or AD2 (**B**), concentration of C1q mimetics ranged from 0 to 20 µg/mL and sC1q was used at a constant concentration of 10 µg/mL. The 0 µg/mL mimetic value was used as a reference for normalization for each IgM separately. Standard deviations are generated from two individual assays.

Figure 3 shows that increasing C1q mimetic concentrations mixed with a constant concentration of sC1q (10 µg/mL) results in decreased sC1q signals. For all four IgM coating variants, inhibition was achieved with both C1q mimetics. Complete displacement of sC1q could not be achieved but at least 59% of sC1q displacement was possible with ACB, and 23–47% inhibition of sC1q binding was enabled with AD2 (Figure 3, Table 1).

For ACB in combination with the 6IgM 2G12 (Figure 3A, light orange-colored blocks), the highest inhibition (77%) of sC1q binding could be reached, but already a 50% reduction in sC1q binding was achieved with 1.3 µg/mL ACB, while higher concentrations of ACB (2.5–20 µg/mL) only slightly improved inhibition (67–77%). With the 5IgM 2G12 (Figure 3A, dark orange-colored blocks), 50% inhibition was observed with a concentration of 5.0 µg/mL of ACB. Similar results were seen for HB617. A 50% inhibition was achieved with 1.3 µg/mL of ACB for the 6IgM and, in the case of 5IgM, the 50% displacement of sC1q was accomplished with 2.5 µg/mL of ACB (Figure 3A, green-colored blocks). Taken together our data suggested that the interaction between 6IgMs and sC1q could be inhibited by slightly less ACB compared to 5IgMs. This may indicate the looser binding of 6IgM HB617/sC1q or the more effective binding of ACB to 6IgM HB617.

The Inhibition potential of AD2 was lower compared to ACB and could not reach 50% inhibition. The highest values were 47% at 2.5 µg/mL of AD2 with the 6IgM 2G12 and 43% at 5 µg/mL of AD2 with the 6IgM HB617, while inhibition reached 39% at 5 µg/mL of AD2 with the 5IgM 2G12 and 23% with 2.5 µg/mL of AD2 at 5IgM HB617 (Figure 3B, Table 1).

In addition to the maximal inhibition of the C1q mimetics, relative IC_50_ values were calculated for each IgM curve for both C1q mimetics. For this calculation, we normalized the inhibiting potential of C1q mimetics and defined the highest C1q mimetic concentration as 100% for each experimental setup. These values are presented in Table 1. The data indicate that C1q mimetics have a greater displacement potential when combined with hexameric IgMs, particularly for HB617, compared to pentameric IgMs.

To estimate the interaction stoichiometries, we calculated the molecular ratios by considering the molecular mass of sC1q and ACB. For instance, when using 1.3 µg/mL of ACB, we achieved 50% inhibition of 10 µg/mL of sC1q with hexameric IgM coating. This corresponds to the 1.2 molecules of ACB required to displace one molecule of sC1q with six binding sites for the IgM molecule.

### 3.4. Complement Activation by IgM

**Figure 4:**
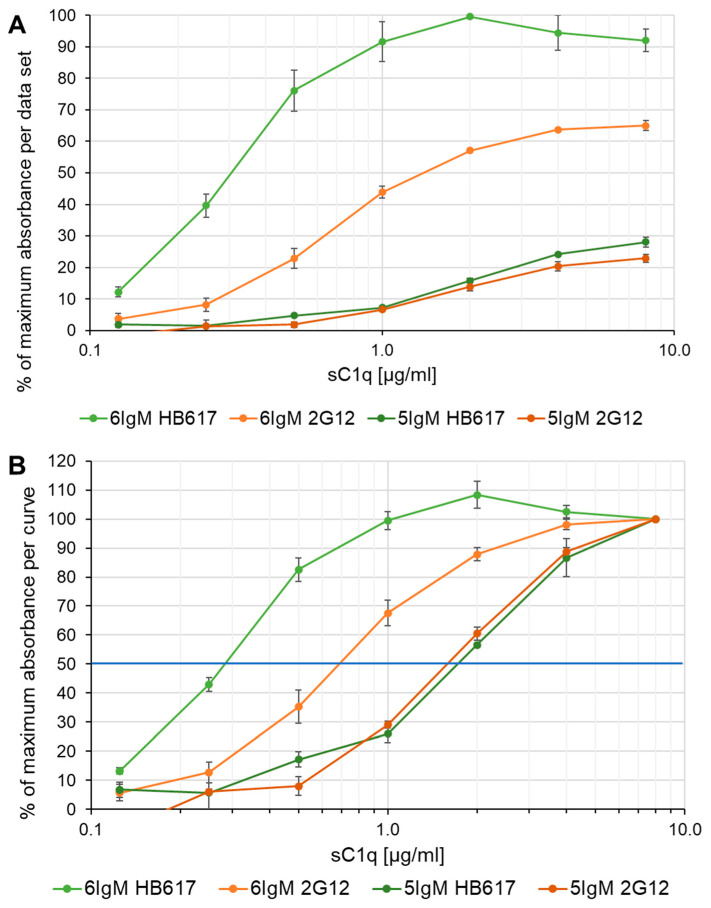
Complement activation assay with IgM coating and C4b readout. (**A**) Percentage of the absorbance normalized to the highest absorbance value obtained within one data set. (**B**) Percentage of the absorbance normalized to the value of the highest sC1q concentration of each IgM coating. Blue line marks the 50% of the maximum absorbance. Standard deviations are calculated from two individual experiments.

To quantitatively evaluate the functionality of the recombinant IgM/sC1q interaction and the competition with recombinant C1q mimetics, a standard 96-well complement activation assay with C4b deposition readout was used [24,27,28]. Plates were coated with hexameric or pentameric IgMs with both specificities, HB617 or 2G12. C1q-depleted normal human serum complemented with sC1q serial dilution was used as the complement source. Figure 4 shows data from the same experiment using different evaluation and display methods. To compare the different IgMs coated on the plate, the absorbance values were normalized according to the highest signal reached with the coating 6IgM HB617 (Figure 4A). The highest value for the 6IgM 2G12 was 65% indicating a weaker C4b deposition for this IgM specificity. The maximum C4b readout of both pentameric IgMs was only about 25%. Differences in the initial slope of the IgMs were also observable. The 6IgM HB617 curve started with a steep increase at low sC1q concentrations and reaches a plateau at 1 µg/mL sC1q. Both hexameric IgM coatings generated a sigmoidal curve shape, while the pentameric IgM curves showed a rather slow and flat course.

In Figure 4B, each curve was normalized to the absorbance value of its highest sC1q concentration (8 µg/mL). This allowed 50% of the maximum absorbance to be plotted and the effective concentration (EC_50_) to be estimated (blue line, Figure 4B). The EC_50_ for 6IgM HB617 was reached at 0.3 µg/mL sC1q, for 6IgM 2G12 at 0.7 µg/mL, for the pentameric HB617 at 1.3 µg/mL and 2G12 IgMs at 1.4 µg/mL.

The activation potential of the IgMs, stated by the EC_50_, thus differed significantly between pentameric and hexameric configurations. The differences between the two configurations were smaller for 2G12 compared to HB617. Specifically, the hexameric HB617 IgM coating had an activation potential that was 4.6 times higher than the pentameric configuration. For the hexameric 2G12 IgM, the calculated activation potential was 2.1 times higher than for the pentameric 2G12 IgM.

The complement activation assay with the two evaluation methods leads to two key findings. Firstly, hexameric IgMs required a lower sC1q concentration to activate the complement compared to pentameric IgMs. Secondly, despite containing the same quantity of IgMs being coated on the plate and sC1q available in the well, more C4 was cleaved upon 6IgM binding compared to 5IgM binding. Notably, these major differences between hexameric and pentameric IgMs were only slightly observable in the IgM/C1q interaction assay (Figure 2).

### 3.5. Complement Activation by IgM and Inhibition by C1q Mimetics

As C1q mimetics do not carry the C2r2s binding/activation region, they lack the potential to cleave C4. Therefore, the functional binding of ACB and AD2 to IgM was assessed in a competitive manner using the same settings as above. Plates were coated with 5IgM HB617 and sC1q was used as sample together with an 8- or 16-fold excess of the C1q mimetic ACB as an inhibitor. C1q-depleted normal human serum was used as the complement source.

**Figure 5:**
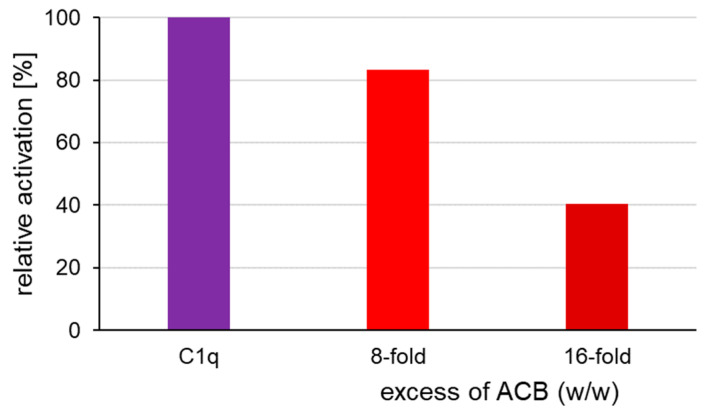
Competitive complement activation with 5IgM HB617 coating, ACB inhibition and C4b readout.

Figure 5 shows that in order to detect inhibition of the C1q mimetic ACB in the competitive activation assay, the sC1q:ACB ratio must be weighted more towards the ACB side than in the competitive interaction assay (Figure 3). An eight-fold (*w*/*w*) excess of ACB resulted in a 20% reduction in C4b deposition and a 16-fold (*w*/*w*) excess was able to reduce C4b deposition by 60% (Figure 5). Calculated in molar ratio, an eight-fold excess of ACB represents a 13-fold excess of globular C1q knobs and a 16-fold of ACB excess displays a 26-fold excess of globular C1q binding sites.

### 3.6. Analysis of Protein Interactions Measured by Bio-Layer Interferometry

Protein–protein interactions were measured by bio-layer interferometry (BLI) using Protein L tips as in Chouquet et al. [24]. BLI tips were loaded with two different hexameric or pentameric IgMs and association and dissociation of sC1q, ACB and AD2 were measured. A global fit and a fast 2:1 heterogeneous ligand model were applied to evaluate k_on_, k_off_ and K_D_ values of the IgM/sC1q interaction. Although this model assumes analyte binding at two independent ligand binding sites, it is the only model available so far to interpret the complicated behaviors of the binding kinetics of the IgM/C1q complex.

**Figure 6:**
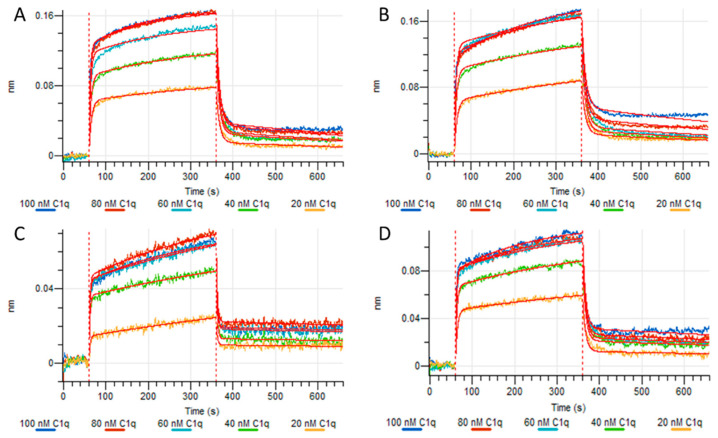
Octet measurements of different IgMs loaded on Protein L tips with sC1q. (**A**) hexameric IgM HB617, (**B**) hexameric IgM 2G12, (**C**) pentameric IgM HB617, (**D**) pentameric IgM 2G12.

As shown in Figure 6, the signals obtained from the sC1q interaction with the IgM ligands were relatively low, which has been previously observed by Chouquet et al. [24]. For both hexameric IgMs, HB617 and 2G12 (Figure 6A,B), sC1q association results in a higher signal than for their corresponding pentameric IgM loads (Figure 6C,D). For all IgM loads, the curves of the three highest sC1q concentrations were very similar, indicating that the maximum response had already been attained with the applied concentrations.

Table 2 shows the calculated on and off rates from the global fit and the fast 2:1 heterogeneous ligand model. Altogether, sC1q association and dissociation kinetics rates were in the same range, leading to affinities in the ten nanomolar range. In particular, dissociation rates (k_off1_) appeared to be faster for hexamers than for pentamers leading to a lower apparent affinity of sC1q for the higher oligomeric states. The 5IgM HB617 appeared to be a special case as the calculated affinity constant, K_D2_, differed significantly from the other IgMs, being the result of a faster k_off2_ and a slower k_on2_.

**Figure 7:**
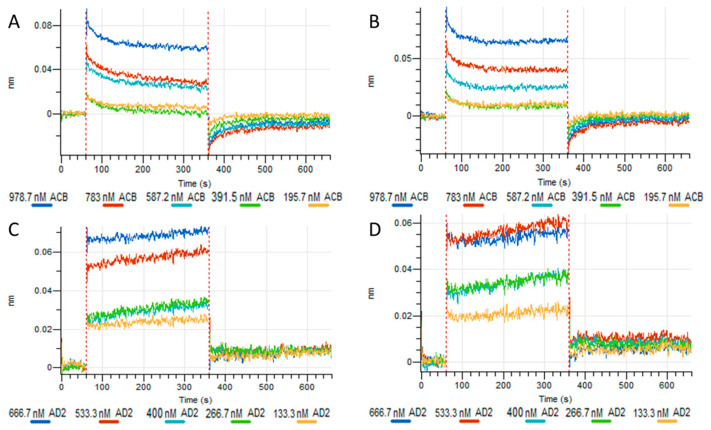
Octet measurements of hexameric and pentameric HB617 IgMs loaded on Protein L tips with ACB or AD2. (**A**) hexameric IgM HB617 and ACB, (**B**) pentameric IgM HB617 and ACB, (**C**) hexameric IgM HB617 and AD2, (**D**) pentameric IgM HB617 and AD2.

In the next experiment, we assessed the interaction kinetics of ACB and AD2 with only the hexameric and pentameric HB617 configurations. The results from Octet measurements depicted in Figure 7 demonstrate that the C1q mimetics bound fast to the captured IgM ligands. Furthermore, it seems that the binding behavior of the two C1q mimetics is affected by the albumin fusion partner. Figure 7A,B show ACB as the analyte, while in Figure 7C,D AD2 was measured.

ACB rapidly bound to both hexameric and pentameric IgMs (Figure 7A,B), and the binding signal slightly decreased before reaching a stable signal. During dissociation, the signal dropped below zero for both IgM conformations. The lower and upper limits of the analyte were indicated by overlapping curves as for 5IgM/ACB and 5IgM/AD2.

The two lowest concentrations of ACB overlapped, while the higher concentrations showed stronger separation with a higher signal during association. This overlap suggests that the two lowest concentrations represented the lower limit of interaction detection.

For AD2 as analyte (Figure 7C,D), an immediate and fast initial association was also observed and dissociation occurred immediately after the buffer change with signal remaining above zero for both IgM loadings. The two highest concentrations, 666.7 nM and 533.3 nM, during association phases reached similar signals, indicated the upper limit of interaction detection.

A comparison of the interaction kinetics between sC1q and C1q mimetics with IgM thus revealed differences in their binding behavior, as evidenced by the association and dissociation curves. Indeed, the BLI data suggest a K_D_ value in the µM range for the IgM/C1q mimetic interaction, as indicated by the limited fitting of the C1q mimetics with the R_max_ value versus concentration in preliminary experiments. To summarize, the interaction between IgMs and sC1q displayed K_D_ values in the nM range, while the interactions between IgMs and sC1q mimetics were determined by a fast on and off rate and low affinities in the µM range.

## 4. Discussion

In this study, interactions of different IgMs with serum-derived C1q (sC1q) and C1q mimetics were analyzed. To characterize the differences between the IgMs in their binding abilities of sC1q and new C1q mimetics, ELISA and bio-layer interferometry (BLI) were both used and four IgMs differing in their specificities and their configurations were employed. The two specificities were HB617 or 2G12 directed against the sphingolipids GM3/GD3 of tumor cells or the HIV gp120 protein, respectively. For each specificity, the recombinantly expressed hexameric (without joining chain) and pentameric (with joining chain) configurations were used. Chen et al. [33] showed that the joining chain might cause asymmetric behaviors of the Fabs of IgM when bound to the antigen compared to the symmetric hexameric IgM, which would then affect C1q binding and complement activation. Therefore, the two recombinant IgMs are referred to as two different configurations. sC1q was used for method development and the two single-chain C1q mimetics were designed, expressed, and purified for characterization with the IgMs.

The interaction assays in ELISA format with the four coated IgMs and sC1q as analyte revealed only minor differences between the different specificities and configurations of the IgMs (Figure 2). In contrast, the difference, especially between hexameric and pentameric configurations of IgMs, was clearly observable with the complement activation assay, where the hexameric IgMs triggered the complement at least twice as efficiently as pentameric IgMs (Figure 4). We hypothesize that this occurs due to the different setups of the tests. The binding assay records a distinct point in the equilibrium of the on/off binding kinetic of IgM and C1q and detects this via an anti-C1q antibody. The activation test provides information on the accumulation of cleaved C4b during incubation when sC1q is bound and the complement cascade is activated [34].

Sharp et al. [6] stated that pentameric IgM-C1 structures may contain one or two C4b molecules, while hexameric IgM-C1 structures were observed with two bound C4b molecules. This might lead to greater differences in complement activation assay results between hexameric and pentameric IgMs, but less pronounced in interaction assays.

In the study by Hennicke et al. [28], two out of the four IgMs used in this study (pentameric HB617 and pentameric 2G12) were applied for coating, and complement activation has been performed with C1q-depleted normal human serum reconstituted with 4 µg/mL of sC1q. No significant differences were observed between the different specificities. This is in agreement with the data obtained in our study, as the differences between the specificities regarding the activation potential of the IgMs could only be demonstrated for the hexameric IgMs, whereas the pentameric IgMs showed only minor differences (Figure 4).

In the study by Chouquet et al. [24], hexameric IgMs of both specificities have been also used in the same assay set up with a single C1q concentration (4 µg/mL). No significant differences were observed between the different specificities, or between pentameric and hexameric IgMs. The dilution series of sC1q used in our study covered a wider range of the sC1q-dependent complement activation potential of the different IgMs and therefore, the differences between pentameric and hexameric configurations could be observed, especially at lower sC1q concentrations. Our findings show that the hexameric configuration presented a 2.1–4.6 higher activation potential than the pentameric configuration, which is consistent with Collins et al. [35]. In their study, complement activation was assessed by complement-dependent lysis of erythrocytes, and hexameric IgM has been found to activate approximately 3–13 times more efficiently than pentameric IgM. The differences in complement activation between hexameric and pentameric IgMs result from the structural formation of the IgM-C1 complex This is either described by the binding capacity of hexameric IgM/C1 complex for C4b molecules, as previously mentioned, and, additionally, may be related to the different non-planar, dome-shaped structures observed when bound to a surface or an antigen [6]. While the hexameric IgM forms a stable hexagonal structure, the pentameric IgM can be described as an asymmetric hexagon, with the sixth IgM monomer being replaced by the joining chain [36,37,38,39].

A study by Chouquet et al. [24], which focused on the biophysical characterization of recombinant IgMs and their C1q binding kinetics by BLI, has already provided data on two of the IgMs which were also used in this study (IgM617 CHO DG44 = 5IgM HB617, IgM012 CHO DG44 = 5IgM 2G12). They reported minimal differences in kinetic rates between their recombinant IgMs expressed with and without joining chain but were rather careful in their assessment since these variations were also observed between different pentameric IgMs. The higher k_off1_ value observed for the hexameric IgMs in this study was not observed. In general, all K_D_ values of the IgM/sC1q interaction vary in the nM range (Table 2).

The affinity of C1q to IgM as measured by BLI did not significantly differ between the four IgMs neither in the binding curves (Figure 6) nor in the calculated affinity (Table 2). We suggest that additional highly complex interaction mechanisms are responsible for the differences in the complement activation [6,33].

The C1q mimetics ACB and AD2 imitate the globular head of the C1q protein omitting the collagen-like regions. To facilitate expression, the globular domains were connected via short linkers and expressed as single-chain proteins. A study by Moreau et al. [14] compared the crystal structures of the decollagenated serum-derived C1q globular domain and their recombinant single-chain variant of the globular head [40]. The global superposition demonstrates that they are almost identical. Consequently, the expression of the C1q globular head as a single-chain variant appears to be a validated approach for mimicking this part of C1q.

The literature provides only limited information regarding the interaction between IgMs and single-chain C1q mimetics. Vadászi et al. [11] conducted a study on a protein construct expressed in *Escherichia coli* that is similar to ACB and used surface plasmon resonance (SPR) measurements to evaluate a K_D_ value in the nM range as we observed for sC1q. In a study by Moreau et al. [14], interactions of a single-chain version of the globular region of C1q, serum-derived globular region of C1q and serum-derived C1q with different physiological ligands of C1q were also analyzed by SPR. The C1q globular domain proteins exhibited a lower binding affinity, as evidenced by measured K_D_ values that are 24–48 times higher compared to the full-length C1q. Our observation is consistent with their findings that the association and dissociation of the C1q mimetics occur more rapidly than those of sC1q, which can be explained by the diminished binding propensity with the missing avidity of the single-chain mimetics compared to the entire hexameric C1q molecule. In general, interaction studies of IgM and C1q mimetics are limited by the K_D_ value in the µM range. By definition, measured analyte concentrations should be up to 10 times the expected K_D_ [41]. This implies that the highest concentration of ACB and AD2 in BLI measurement should be 10 times higher than the concentration actually applied. The stacked curves of the two highest concentrations during the association of AD2 to IgMs in Figure 7, panels C and D, suggest that these recommended high concentrations are unnecessary.

The calculation of the binding site ratio in the competitive IgM-C1q interaction assay indicated that the C1q binding to IgM is already diminished by 50% with the hexameric IgM coating when only one or two binding sites are occupied by the C1q mimetic ACB. We hypothesize that despite the fast association and dissociation of the C1q mimetics, the binding of the globular C1q heads of the whole C1q could be disturbed and the binding of C1q is inhibited.

Vadászi et al. [11] expressed a protein similar to ACB in *Escherichia coli*. They conducted a competitive activation assay with IgM coating and C4 detection, and found 100% inhibition. However, their assay setup included a higher concentration of the coated IgM (40 nM vs. ∼10 nM) and a higher sample concentration (2000 nM vs. 618 nM ACB). As whole serum was used as the source of C1q, the exact amount of C1q available in the assay could not be determined. Therefore, the results suggest that 100% inhibition can be achieved with an assay setup including higher protein concentrations.

Noteworthily, this inhibitory effect of C1q mimetics is evident predominantly in the competitive interaction assay (Figure 3), but only to a minor degree in the competitive activation assay (Figure 5). One explanation can be the methodological approach with an accumulation of C4b binding due to enzymatic cleavage in the course of C1 activation during the incubation period. Secondly, we hypothesize that the IgM-C1q binding might be stabilized by the entire C1 complex and is therefore less disturbed by the C1q mimetics. This leads to the low inhibitory effect of activation by C1q mimetics.

## 5. Conclusions

In this study, we demonstrated that recombinant IgMs, expressed in both pentameric and hexameric configurations and with two specificities, exhibit comparable interaction with sC1q. However, our investigation concerning complement activation revealed a notable disparity in C4b deposition between hexameric and pentameric IgM variants, with hexameric IgM displaying an over twofold higher activity. This observation was substantiated by both signal intensity and the calculated EC_50_ values. Furthermore, our findings indicate that both recombinant C1q mimetics, ACB and AD2, effectively inhibit the interaction between IgM and C1q, as well as subsequent complement activation.

Our study underscores the significance of structural configuration and specificity of IgMs in modulating IgM/C1q interactions and subsequent complement activation. The observed differences between hexameric and pentameric IgM variants, as well as between specificities, highlight the nuanced nature of these interactions. Furthermore, the efficacy of C1q mimetics in inhibiting IgM/C1q interaction suggests their potential utility as tools for probing this interaction and warrants further investigation. The recombinant expression of a single-chain mimetic of the globular C1q head appears to be a valuable approach to the analysis of specific protein–protein interactions. A more advanced insight into the protein–protein interaction could be achieved by random mutagenesis libraries and selection of high-affinity binders and non-binding mutants.

The design of an easier-to-express protein to mimic a difficult-to-express protein paves the way for the performance of library research and the identification of mutations with the objective of achieving a more advanced insight into protein–protein interaction behavior.

Overall, our findings provide valuable insights into the molecular mechanisms underlying IgM/C1q interactions and complement activation. Furthermore, our study paves the way for future research aiming to develop recombinant C1q variants for scientific inquiry and clinical applications.

## Figures and Tables

**Table 1:** The maximal inhibition of the C1q mimetic and the relative IC_50_ values of the competition ELISA with IgM coating and anti-C1q detection. The highest C1q mimetic value was used as 100% for the relative IC_50_ values, and the OD value with no mimetic was used as 0% inhibition for each IgM coating. The actual tested C1q mimetic value, which was close to 50%, is indicated.

IgM/C1q Mimetic	Maximal Inhibition of ACB [%]	Maximal Inhibition of AD2 [%]	Relative IC_50_ ACB [µg/mL] (Approximate Values)	Relative IC_50_ AD2 [µg/mL] (Approximate Values)
6IgM HB617	68	43	0.6	0.3
6IgM 2G12	77	47	1.3	0.6
5IgM HB617	70	39	1.3	1.3
5IgM 2G12	59	23	1.3	1.3

**Table 2:** K values of the protein interaction between IgM and sC1q.

IgM/sC1q	k_on1_ [10^4^/Ms]	k_on2_ [10^6^/Ms]	k_off1_ [10^−4^/s]	k_off2_ [10^−1^/s]	K_D1_ [10^−9^ M]	K_D2_ [10^−9^ M]
6IgM HB617	6.4	2.1	14.3	1.3	22.1	61.5
6IgM 2G12	3.3	2.0	12.5	1.1	37.9	54.3
5IgM HB617	3.1	1.4	2.8	3.7	9.3	271
5IgM 2G12	5.0	3.6	6.2	1.4	12.5	39.6

## Data Availability

The original contributions presented in the study are included in the article, further inquiries can be directed to the corresponding author.
